# Supplementary material for: Cell Identity Codes: Understanding Cell Identity from Gene Expression Profiles using Deep Neural Networks
Source: Sci Rep. 2019 Feb 20;9:2342. doi: 10.1038/s41598-019-38798-y (PMC6382891; doi:10.1038/s41598-019-38798-y)

Cell Identity Codes: Understanding Cell Identity from Gene Expression Profiles using Deep Neural Networks

Farzad Abdolhosseini[1,*], Behrooz Azarkhalili[2,*], Abbas Maazallahi[1], Aryan Kamal[1], Seyed Abolfazl Motahari[1], Ali Sharifi-Zarchi[**1] and Hamidreza Chitsaz[**3]

[1]Department of Computer Engineering, Sharif University of Technology, Tehran, Iran
[2]Royan Institute for Stem Cell Biology and Technology, ACECR, Tehran, Iran
[3]Department of Computer Science, Colorado State University, Fort Collins, CO, USA
[*]These authors are equally contributed on this work
[**]Corresponding authors: asharifi@sharif.ir, chitsaz@chitsazlab.org

Supplementing Fig S1. Human phenotypes associated with CIF components.

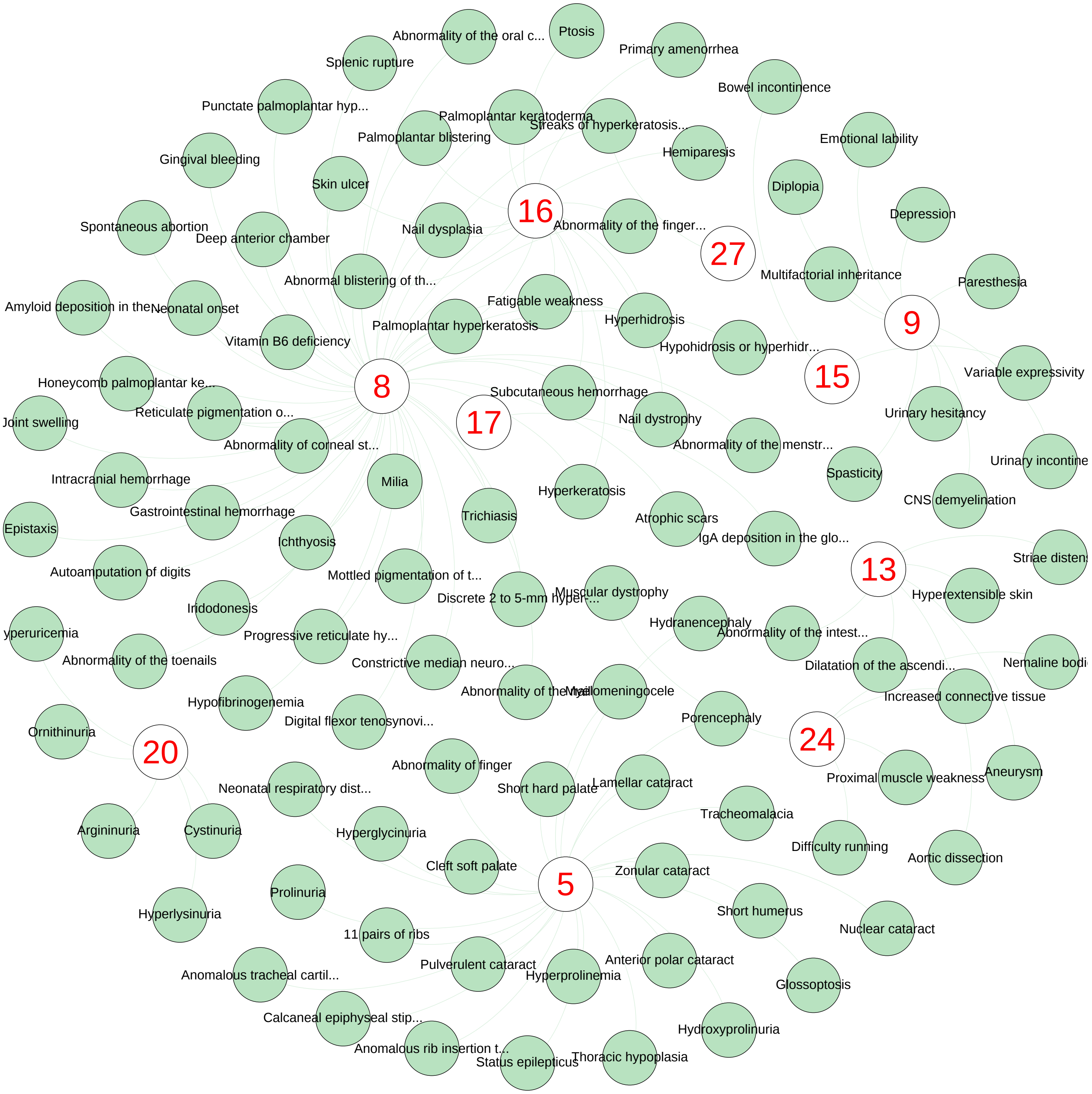

Supplementing Fig S2. GO Molecular Functions associated with CIF components.

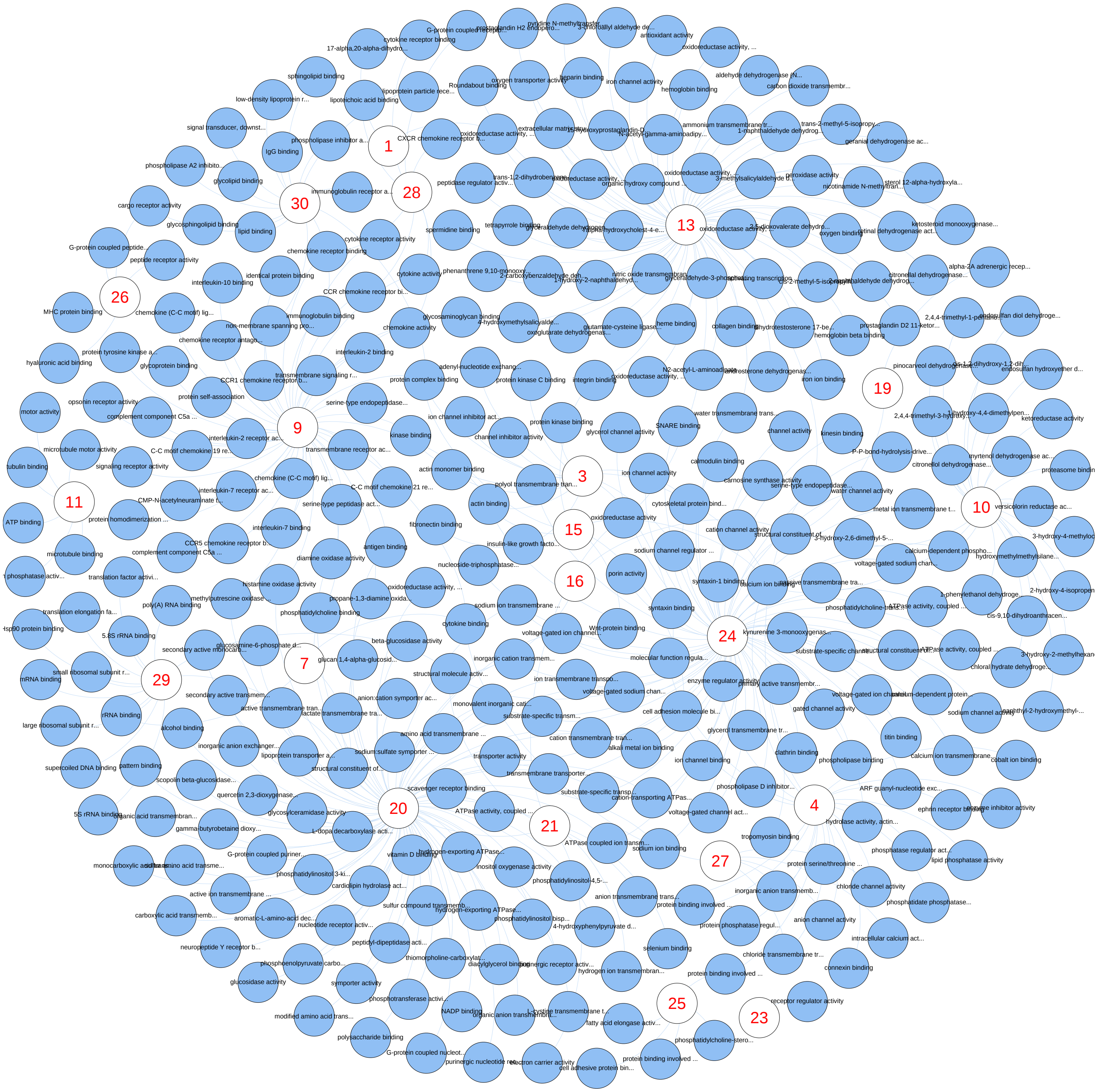

Supplementing Fig S3. Protein Domains associated with CIF components.

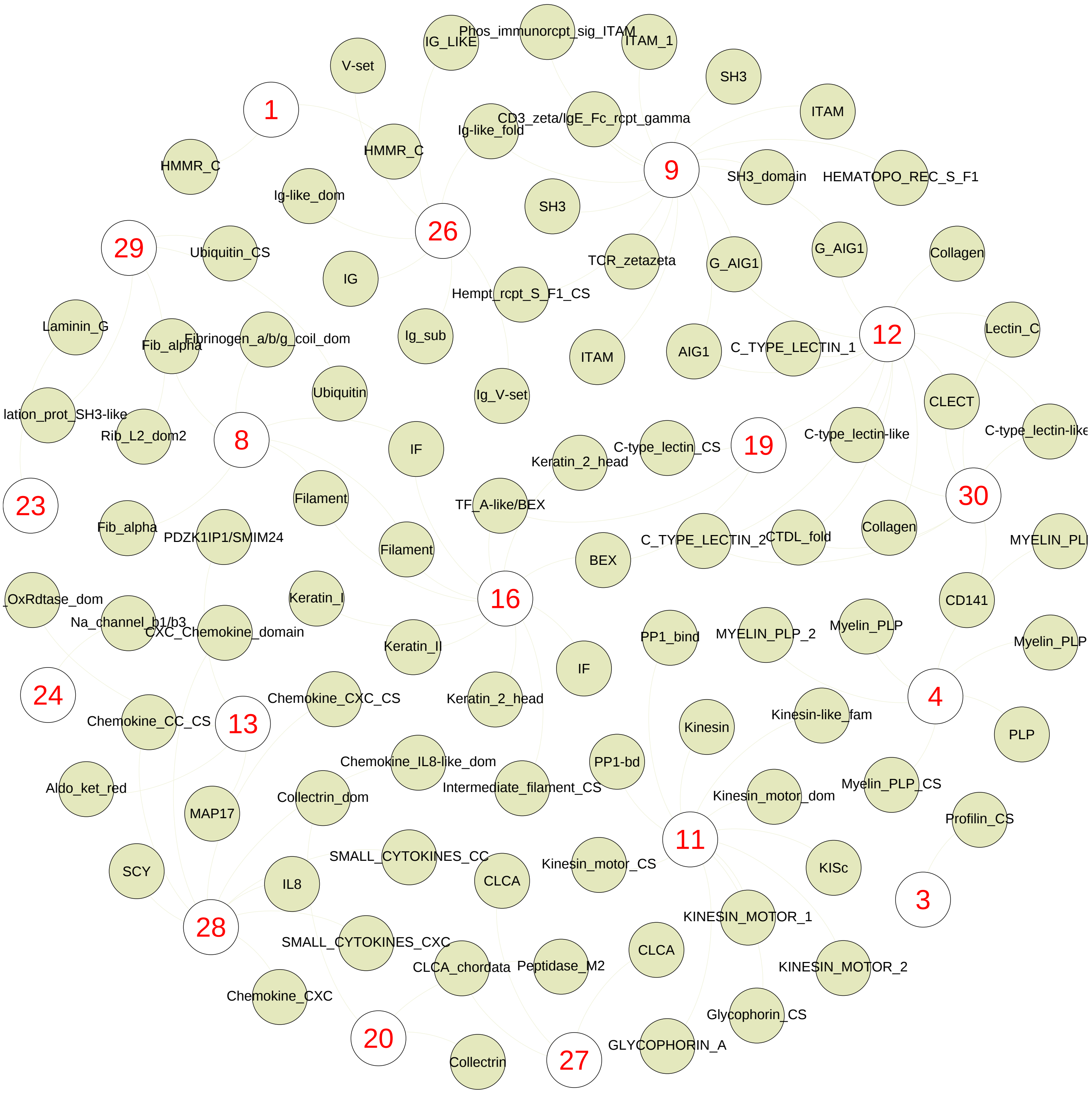

Supplementing Fig S4. MicroRNAs associated with CIF components.

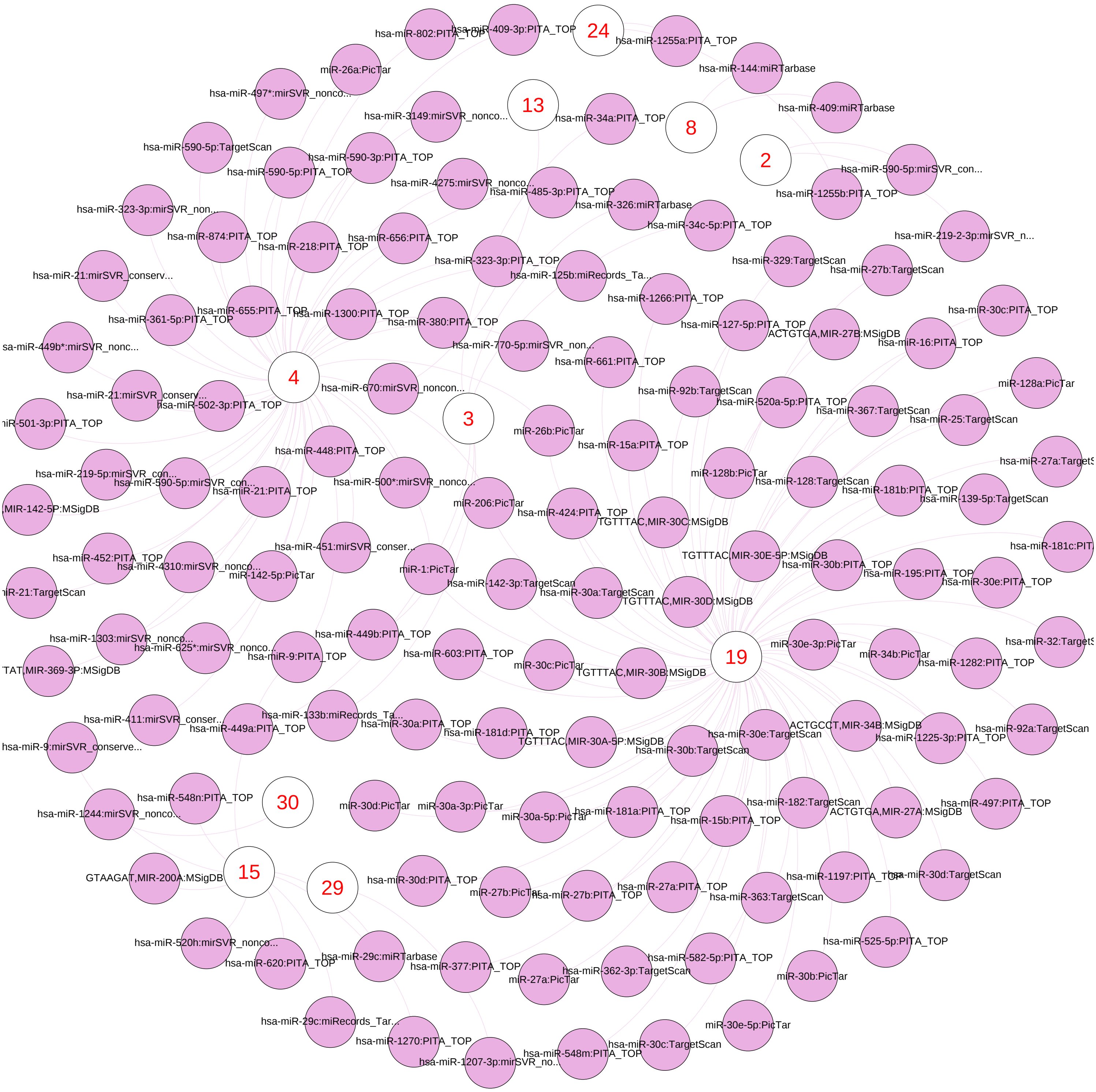

Supplementing Fig S5. Diseases associated with CIF components.

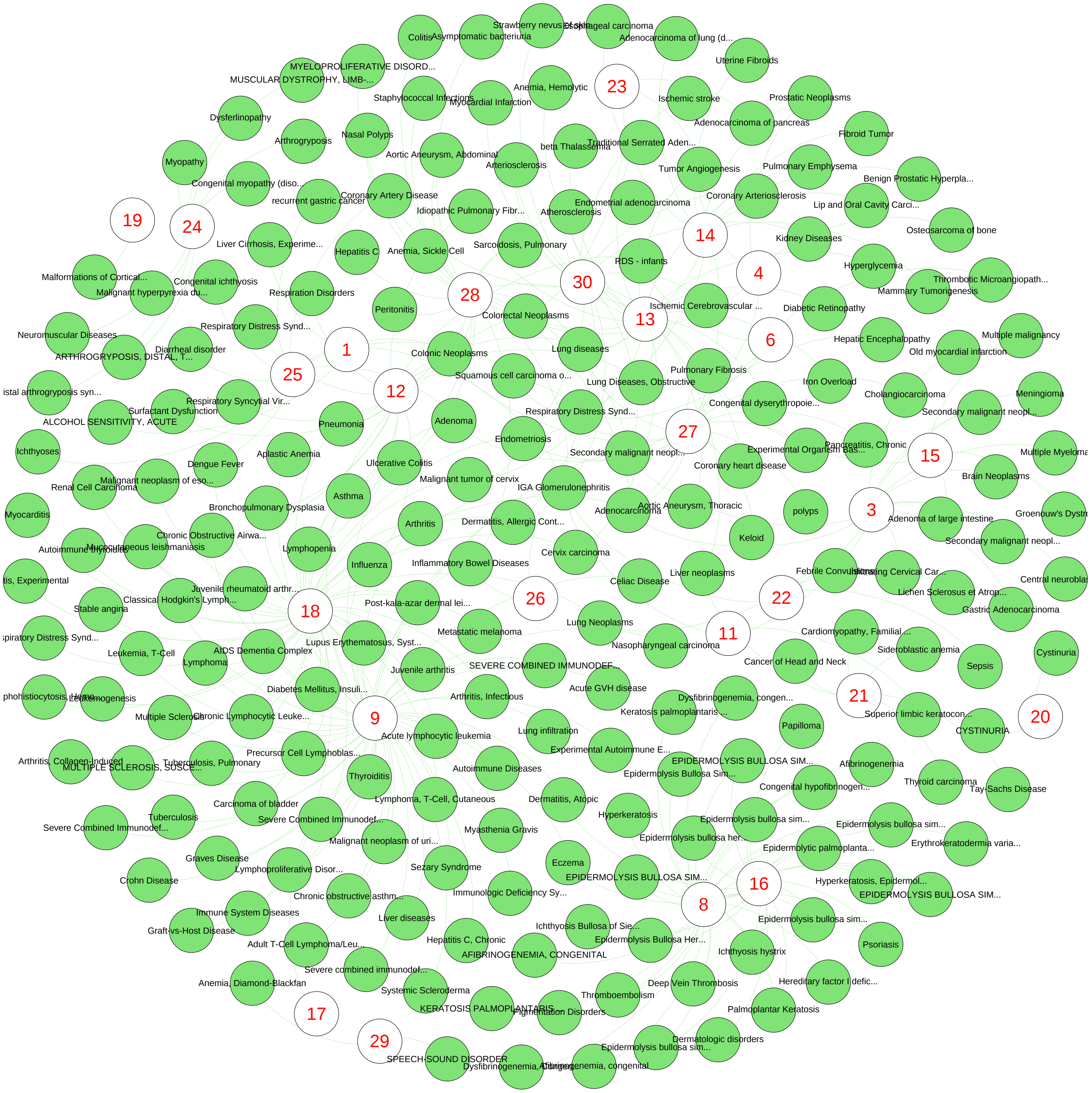

Supplementing Fig S6. Drugs associated with CIF components.

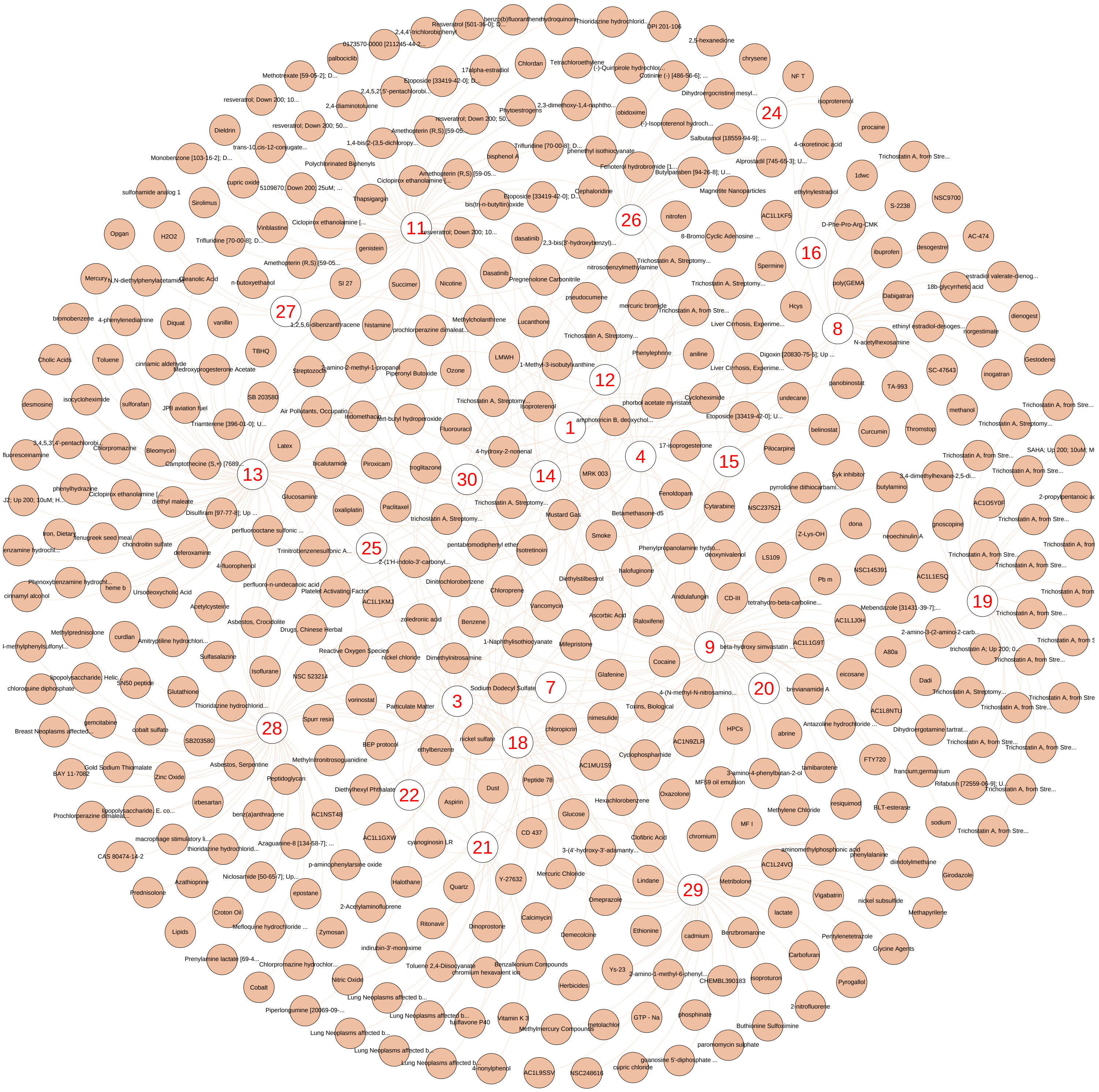

Supplement: Supplementary file 1 — Supplementary figures [file 41598_2019_38798_MOESM1_ESM.pdf]
