# Supplementary material for: Cell Identity Codes: Understanding Cell Identity from Gene Expression Profiles using Deep Neural Networks
Source: Sci Rep. 2019 Feb 20;9:2342. doi: 10.1038/s41598-019-38798-y (PMC6382891; doi:10.1038/s41598-019-38798-y)
Supplement: Supplementary file 2 — Cell Identity Codes: Understanding Cell Identity from Gene Expression Profiles using Deep Neural Networks [file 41598_2019_38798_MOESM2_ESM.pdf]

Cell Identity Codes: Understanding Cell Identity from Gene Expression Profiles using Deep Neural Networks

Farzad Abdolhosseini[1,*], Behrooz Azarkhalili[2,*], Abbas Maazallahi[1], Aryan Kamal[1], Seyed Abolfazl Motahari[1], Ali Sharifi-Zarchi**[1] and Hamidreza Chitsaz**[3]

[1]Department of Computer Engineering, Sharif University of Technology, Tehran, Iran
[2]Royan Institute for Stem Cell Biology and Technology, ACECR, Tehran, Iran
[3]Department of Computer Science, Colorado State University, Fort Collins, CO, USA
[*]These authors are equally contributed on this work
[**]Corresponding authors: asharifi@sharif.ir, chitsaz@chitsazlab.org

Submission of txt file seemed not possible through the system. We converted it to PDF file.
The original source codes, RAW and processed data files are available for submission.

Gene	CIF_Component
PLAC8	1
ANXA3	1
GGH	1
PDLIM1	1
HBB	1
CA2	1
KIT	1
PF4	1
DHTKD1	1
TSPAN13	1
CKS1B	1
RRM2	1
BUB1B	1
LGALS4	1
RAD51AP1	1
TDP2	1
MEST	1
ALDH1A1	1
C5	1
CNRIP1	1
CHAC2	1
E2F8	1
GPR160	1
MMADHC	1
FAM46C	1
DLGAP5	1
KIF15	1
IGJ	1
HHEX	1
NIPSNAP3A	1
MAN1A1	1
EPCAM	1
ZMYM1	1
SERINC1	1
PDZK1	1
CMPK2	1
PCK2	1
COPZ1	1
TSPAN8	1
STARD3NL	1
BMP2	1
CHMP4C	1

IMPA2	1
DEPDC7	1
LTA4H	1
ALDH2	1
RNF128	1
BLNK	1
BORA	1
APOBEC3B	1
STARD4	1
HCLS1	1
ITGB3BP	1
ADM	1
RNASE6	1
ZFP112	1
PEX11A	1
GNG5	1
PCNA	1
GOLGA8H	1
KIAA0125	1
CHEK1	1
TBCK	1
SPIN4	1
PHYH	1
OSTC	1
PRKAR2B	1
MFAP3	1
EBPL	1
HSD17B2	1
FZD5	1
ACADM	1
ETFDH	1
KHNYN	1
HMMR	1
PLS1	1
TIGD2	1
PAPSS2	1
PSMA2	1
SFTPC	1
VAMP7	1
SLC25A43	1
IGFBP7	1
LRRC19	1
SERPINH1	1
GIN1	1
KRT18	1

ZCCHC7	1
HMCN1	1
SMARCC1	1
DTL	1
SCP2	1
ANKMY2	1
HSD17B11	1
CETN3	1
AKR1C3	1
PXMP2	1
BTK	1
CXXC5	1
KTN1	1
C7orf23	2
COMMD8	2
ATP6V1E1	2
USP9Y	2
C14orf142	2
ATP6V1A	2
ITGB1BP2	2
RPS4Y1	2
GPD1L	2
PRKACB	2
DPY19L2	2
KIF3A	2
TTC1	2
EIF2A	2
EIF2C3	2
AMD1	2
LDHB	2
STK39	2
FASTKD3	2
LOC150381	2
NSRP1	2
PFKM	2
NAP1L5	2
LYPLAL1	2
SNHG12	2
BEX4	2
C12orf75	2
SLC25A5	2
DSC1	2
MRPL1	2
EFTUD1	2
PAM	2

TYRP1	2
WDR12	2
ZBTB8A	2
FHL1	2
LOC100506165	2
MAL	2
KIF9	2
COPS5	2
WDR47	2
UBR7	2
ASUN	2
GIMAP4	2
FAM96B	2
LAPTM4B	2
PPA1	2
CDC42EP3	2
ZDHHC6	2
IL7R	2
LOC729852	2
ARHGEF6	2
B3GALNT1	2
CHMP3	2
TIPARP	2
CRIP2	2
SLC16A7	2
FZD6	2
EPRS	2
MRPL3	2
ZNF195	2
CRY1	2
DGKI	2
CAB39L	2
FABP5	2
SLC39A6	2
CCDC99	2
COX7A1	2
MPHOSPH9	2
ITM2A	2
NMD3	2
DHX36	2
GHITM	2
EID2	2
PKIA	2
EPHA4	2
PMP22	2

DNAJB14	2
PAICS	2
CRYAB	2
CHN1	2
SELT	2
RNF150	2
BOLA3	2
CHCHD1	2
DNAJA4	2
TPR	2
SPARCL1	2
LOH12CR1	2
ARMCX1	2
NARS	2
ITK	2
MCCC1	2
CLDND1	2
SYTL2	2
CWC27	2
ATP2B4	2
SERINC1	2
GLRB	2
ZBTB11	2
FAM198B	3
GJA1	3
MMP1	3
HSPB1	3
CD63	3
SEPN1	3
SPARC	3
CTGF	3
POLR2J	3
CAPNS1	3
RPL36A	3
ROMO1	3
PLP2	3
TUBB6	3
HN1	3
S100A16	3
CAPG	3
42988	3
CDC42EP5	3
PKM	3
CAV1	3
KCTD12	3

CFL1	3
ACTB	3
PTX3	3
VAT1	3
PDP1	3
MALL	3
IER3	3
CRIM1	3
TMEM98	3
ITGA5	3
ECSCR	3
COL4A2	3
COTL1	3
RPL29	3
SNX7	3
QPCT	3
MARCKS	3
C15orf48	3
ASAP2	3
SPOCK1	3
PFDN5	3
LGALS1	3
PRKCDBP	3
TMCC3	3
DNASE1L1	3
SEPW1	3
TFPI2	3
EIF4G1	3
PLS3	3
FKBP1A	3
PSMB4	3
TMSB10	3
COL5A2	3
CD24	3
PODXL	3
MANSC1	3
DPYSL2	3
LOC375295	3
PROCR	3
TMEFF1	3
CD59	3
SASH1	3
GABARAP	3
PLXNA2	3
PFN2	3

STX7	3
AHNAK2	3
UCHL1	3
TWSG1	3
AP1B1	3
S100A11	3
ALDOA	3
TIMP2	3
PFN1	3
EMP1	3
TP53I3	3
GBAS	3
WLS	3
HHIP	3
MMGT1	3
ARSJ	3
PREX1	3
OAZ1	3
PEA15	3
COPS7A	3
SMAGP	3
RHOJ	3
YWHAZ	3
MAP1B	3
SH3BGRL3	3
CD9	3
VPS28	3
MAP1LC3B	3
PPP2R3A	3
ASNA1	3
CEACAM7	3
PSMD3	3
ITGAV	3
SPARCL1	4
PLP1	4
VSNL1	4
DSP	4
SYBU	4
PTPRZ1	4
PLA2G2A	4
STMN2	4
ODZ2	4
MT1M	4
IGJ	4
WIF1	4

SPINK5	4
PSD3	4
NEURL1B	4
SERPINF1	4
CPE	4
GJA1	4
TSPAN13	4
RCAN2	4
NAP1L3	4
SPHKAP	4
GPM6B	4
KLF4	4
C1S	4
SDR16C5	4
CEACAM6	4
LPPR4	4
CKB	4
RNF128	4
CD24	4
MAL2	4
SLC16A14	4
TMEM56	4
ID1	4
EGLN3	4
FOS	4
PLK2	4
LPHN3	4
HLA.DRA	4
PPP1R14A	4
TSPAN7	4
CDO1	4
CHN1	4
LRRN3	4
APCDD1	4
FXYD1	4
IGFBP3	4
PCDH9	4
PPP1R14C	4
CRYAB	4
ASPN	4
APOD	4
SLC12A5	4
SELM	4
SYTL2	4
CLCA4	4

ST6GALNAC1	4
GRIN2A	4
EFNB2	4
MATN2	4
CLEC3B	4
ZNF415	4
PAIP2B	4
LOC653602	4
UGT2B17	4
ATL1	4
RGS2	4
SCG2	4
FXYD3	4
PEG3	4
DCN	4
KLF5	4
LUM	4
P2RY14	4
EFHA2	4
SERPINI1	4
NDFIP2	4
CUX2	4
EPHA4	4
SNAI2	4
WFDC1	4
SEMA3C	4
CNTN3	4
HLF	4
B3GALT2	4
CXADR	4
GLRB	4
OLFM1	4
GPM6A	4
PPAP2A	4
CEACAM7	4
TCEAL7	4
GNAI1	4
MAMDC2	4
HBB	4
SLC6A1	4
EGR3	4
HMP19	4
CDH1	4
CDK5	5
UBE2S	5

P2RX5	5
RNASEH2A	5
RBM38	5
MND1	5
SNRPB	5
TTYH2	5
TFPT	5
SYNGR3	5
42979	5
YPEL4	5
RUNDC3A	5
PRKCD	5
KCNN4	5
MAL	5
LIMD2	5
CHST2	5
RAB6B	5
CENPM	5
BEX2	5
BEX1	5
SAPCD2	5
NEIL1	5
GBF1	5
CENPW	5
CDC20	5
RAD9A	5
GNG3	5
TUBG1	5
DEDD2	5
DDX49	5
FERMT3	5
RHBDD2	5
HCFC1R1	5
PLP1	5
CENPH	5
OTUB1	5
NRGN	5
ATG13	5
SH3BGRL3	5
BCL7C	5
CD320	5
KIF2C	5
ATP6V0B	5
FAM98C	5
INA	5

ALKBH6	5
KPTN	5
FARSA	5
C22orf39	5
CA11	5
NPM3	5
CD37	5
PRSS53	5
LOC389023	5
DDX51	5
SLC5A11	5
ACTL6B	5
SELO	5
RBM10	5
PSMG3	5
VASH1	5
SNRNP40	5
POU5F1P3	5
REEP4	5
PIDD	5
BCL11B	5
DLGAP5	5
C6orf1	5
DAPK3	5
RHOBTB2	5
C20orf27	5
RFWD3	5
POLE	5
HSF4	5
HBQ1	5
XAB2	5
TPX2	5
MRPL21	5
GPR56	5
PTPRZ1	5
PRODH	5
ISG20	5
RAB5C	5
SLC6A8	5
PCLO	5
EPB42	5
ATP8A2	5
CYFIP2	5
PIN1	5
TDRKH	5

NAT6	5
GPR160	5
FAM100A	5
PRR12	5
C7orf59	5
STEAP1B	5
PROK1	5
CALB1	5
RCSD1	6
TOX2	6
FAM46C	6
COL1A2	6
COL6A3	6
ARHGEF6	6
ICAM3	6
LUM	6
PPBP	6
IGJ	6
FOSB	6
NLRC3	6
P2RY8	6
PTCSC1	6
FBLN2	6
ISLR	6
SFTPB	6
MPEG1	6
CD69	6
CRP	6
MAMDC2	6
C1S	6
CPB2	6
ARHGAP9	6
SFTPC	6
LOC643733	6
ZNF37BP	6
SFTPA2	6
CPS1.IT1	6
KIAA0355	6
C10orf47	6
C6orf70	6
LRRN1	6
GLI4	6
CA3	6
TSPAN2	6
SFTA3	6

LOC100132741	6
NLRC5	6
IFI44L	6
FCRLA	6
TCIRG1	6
PKHD1L1	6
ZNF768	6
C16orf54	6
BACH2	6
GIMAP2	6
CBFA2T3	6
ZSCAN29	6
ADH1B	6
IRF7	6
LBH	6
PPP1R26	6
AGBL2	6
FOXF1	6
APOA2	6
CYP3A4	6
EPB41L5	6
CIRBP	6
TMOD4	6
GIMAP7	6
KAL1	6
GZMH	6
AEBP1	6
SAT2	6
KIAA1652	6
TNNC1	6
ITGB2.AS1	6
CACNA2D2	6
MIR214	6
THAP11	6
HMHA1	6
TCL1A	6
PPP1R14A	6
LOC100132999	6
CMYA5	6
TK2	6
TRIM22	6
C1QTNF1	6
ABCB4	6
IRF8	6
TMEM182	6

ECM2	6
SVEP1	6
CXorf21	6
SELL	6
KLF2	6
GSTM2	6
ZMYM5	6
KCTD11	6
VEGFC	6
GHR	6
STAP1	6
MGC27345	6
42979	6
TAT	6
LOC553103	6
WNT5A	6
CNKSR1	6
EFHA2	6
IFITM1	7
S100A4	7
ITK	7
NPM1	7
PFKFB3	7
RASGRP1	7
CHSY1	7
AMPD2	7
VOPP1	7
ADPRHL2	7
GBP1	7
RRM2	7
GBP2	7
EGR3	7
CCR7	7
CD3D	7
TRBC1	7
RPSA	7
DOCK10	7
TMSB10	7
TRIM26	7
OPHN1	7
RPS21	7
IFITM2	7
NLRC5	7
GBAP1	7
CD2	7

WIPF1	7
TNFAIP3	7
RPL39	7
SSTR5	7
TAP1	7
AIM1	7
RPL38	7
ICAM3	7
HLA.E	7
LBH	7
SSR4	7
RAB40B	7
C7orf23	7
RALA	7
HLA.A	7
DUSP2	7
TGFBR3	7
KRT5	7
UBA52	7
RPL28	7
PSME2	7
TBC1D10C	7
FXYD5	7
ITPRIP	7
TNFAIP8	7
TWIST1	7
SCARNA17	7
DSC1	7
TMEM99	7
DENND2D	7
DKFZP586I1420	7
PSMB9	7
COL6A3	7
CSRNP1	7
ANKRD13A	7
KDM2B	7
GPR171	7
PVRIG	7
RPL12	7
CHD4	7
COL1A2	7
HK1	7
CRELD2	7
THBS2	7
ZNF700	7

CDK5RAP3	7
DUSP14	7
RPL3	7
GATA3	7
ADAR	7
IL2RG	7
POLR2G	7
PTPN13	7
ERP29	7
RPL13A	7
CRLF3	7
ICOS	7
RPS18	7
STK39	7
PGAM1	7
CAP1	7
CRIP1	7
LCK	7
HCP5	7
CPSF3L	7
EBNA1BP2	7
LTBP3	7
PLP2	7
GOLGA8B	7
PTMA	7
TCIRG1	7
C20orf24	7
DAPL1	7
DSG1	8
KRT5	8
MRC1	8
AZGP1	8
GPR34	8
HSD11B1	8
DSC1	8
CHP2	8
CSTA	8
FLG2	8
HLF	8
NUDT5	8
KRTDAP	8
SULT2B1	8
IL7	8
TYRP1	8
CFH	8

HPGDS	8
KRT14	8
DEFB1	8
KLK5	8
AKR1D1	8
LRG1	8
HHEX	8
CP	8
PIR	8
FGG	8
DSG4	8
GABRP	8
BAALC	8
FGF12	8
CSF2RB	8
ADRBK2	8
AADACL2	8
MPEG1	8
CBX4	8
DKFZP586I1420	8
ADH1A	8
KRT1	8
PSD3	8
PLD1	8
LTBP3	8
AHNAK2	8
TNFSF13B	8
C11orf80	8
PLXNA2	8
ASPRV1	8
LYVE1	8
IL1R2	8
SYBU	8
IFFO2	8
CYP3A5	8
ZNF57	8
LOC100128252	8
PHYHIP	8
GJB2	8
FGB	8
TMEM176A	8
RYR2	8
C1QB	8
SACS.AS1	8
IFI27	8

FLJ25758	8
NEBL	8
CYP2C19	8
FGA	8
BSPRY	8
ITIH1	8
CFHR4	8
PRKAG2.AS1	8
TTPA	8
CYP4F2	8
PROS1	8
CCR1	8
GGH	8
HSD17B2	8
NGEF	8
HS3ST6	8
PDE2A	8
HPSE	8
AADAC	8
PRSS22	8
TTR	8
RUNDC3B	8
RAB7B	8
CHST13	8
CASP1	8
GNA15	8
KRT8	8
MAL2	8
UGT2B4	8
ODZ2	8
KRTAP4.6	8
NRN1	8
FAM19A2	8
UGT2B17	8
SERPIND1	8
CORIN	8
GLCE	8
MAP4K2	8
CYTIP	9
LAPTM5	9
TRBC1	9
CXCR4	9
AIM1	9
CD52	9
CCL5	9

HLA.DRA	9
CD2	9
CTSS	9
IL10RA	9
LCK	9
SLPI	9
CORO1A	9
GMFG	9
GIMAP7	9
HCLS1	9
TNFSF10	9
S100A9	9
ITK	9
TRAC	9
GIMAP4	9
CCL4	9
GIMAP2	9
TNFAIP3	9
EVI2B	9
RASGRP1	9
SLA	9
PSMB9	9
CD24	9
CCL20	9
IL32	9
LYZ	9
EVI2A	9
IL2RB	9
SELL	9
GZMH	9
LSR	9
IL2RG	9
DENND2D	9
IGJ	9
CD3D	9
IL7R	9
HLA.DPA1	9
MYO1F	9
VAMP8	9
CD8A	9
CCR7	9
PVRIG	9
RASSF5	9
CD14	9
TSPAN8	9

GPA33	9
HLA.DMB	9
SYTL1	9
CD247	9
TYROBP	9
HLA.B	9
C1orf162	9
CD48	9
RARRES3	9
DHRS9	9
CD69	9
LAMP3	9
LST1	9
HCP5	9
BCL2A1	9
HPGD	9
CEACAM6	9
FCGBP	9
FGL2	9
ABP1	9
GZMA	9
HMHA1	9
EFNA1	9
GZMB	9
C1orf116	9
SFN	9
LCP1	9
GPR171	9
ITM2A	9
FXYD3	9
MGAT4A	9
CRIP1	9
HCK	9
HLA.DMA	9
SASH3	9
PTPRC	9
C5AR1	9
TRAT1	9
C16orf54	9
ACSL5	9
C19orf33	9
AGR3	9
IGKV4.1	9
HCST	9
FCER1G	9

RGS1	9
GZMK	9
LSP1	9
ND6	10
XK	10
PROM1	10
MMRN1	10
YWHAG	10
TUSC1	10
TSPYL4	10
ANKRD36B	10
NGFRAP1	10
C3orf80	10
LMO2	10
ALG9	10
PLA2G4A	10
PLOD2	10
MLLT11	10
MEF2C	10
IL18	10
STYK1	10
MGST2	10
AGPAT9	10
HNRNPA3	10
ZNF792	10
C10orf99	10
HOXA5	10
MPO	10
KIAA1211	10
AKR1C3	10
F2RL1	10
PLEKHA6	10
PRSS3	10
WASF1	10
ERG	10
CWH43	10
LINC00342	10
STK39	10
GPSM2	10
EFEMP1	10
MALL	10
RPH3A	10
LOC729680	10
FGD5	10
PRKACB	10

WDR54	10
ZNF521	10
TFEC	10
JAG2	10
RPL38	10
BMX	10
CLU	10
LRP11	10
CALM2	10
NUMB	10
PRTFDC1	10
ASB9	10
GRAMD1C	10
LOC100129502	10
MPP1	10
DHRS11	10
HINT1	10
GJA1	10
FAR2	10
CLCA1	10
CSF2RB	10
SERPINI1	10
SACS	10
DKFZP586I1420	10
MCTP1	10
LOC728093	10
RAPGEF4	10
RPIA	10
SOX7	10
PMP22	10
SOWAHA	10
ASB13	10
CDH5	10
HMG20A	10
CD302	10
LOC100128252	10
EMCN	10
SI	10
RAP1GAP2	10
MDN1	10
ELANE	10
CCDC6	10
GPR56	10
FKBP3	10
COL24A1	10

PSMD14	10
RALA	10
ESAM	10
LOC100507254	10
VDAC3	10
PEAR1	10
SDCBP2.AS1	10
RPS11	10
LOC100506165	10
LARP1B	10
AGR3	10
MRPS25	10
SLC27A2	10
FAM83D	11
AHSP	11
CENPE	11
ITGA4	11
PBK	11
UBE2C	11
TTK	11
SLC25A39	11
UBE2S	11
KIF15	11
TPX2	11
NEK2	11
SHCBP1	11
NUF2	11
CPNE5	11
CD19	11
DKK1	11
HJURP	11
PPP1R15A	11
TSPO2	11
EPB42	11
KIF20A	11
TPST1	11
MCM5	11
HSPA13	11
ISG20	11
DUSP5	11
NT5E	11
KIF18A	11
PSENEN	11
MKI67	11
ALYREF	11

SOCS2	11
LBH	11
NDC80	11
FAM117A	11
BLOC1S4	11
BUB1B	11
RAB39B	11
SLC38A5	11
KIF2C	11
PRC1	11
UBE2M	11
KLF1	11
GYPB	11
PCIF1	11
TK1	11
RETNLB	11
ODC1	11
PSG2	11
SKA1	11
CDCA2	11
PRIM1	11
ID1	11
ASF1B	11
LOC338620	11
TESC	11
MMP3	11
HEMGN	11
FKBP1B	11
DBF4	11
CNR2	11
ST6GALNAC4	11
IRF1	11
RPP40	11
SWT1	11
CDC6	11
TTPAL	11
ASPM	11
SPC25	11
MELK	11
CENPK	11
APOA2	11
NCAPG	11
PTTG1	11
MS4A1	11
RHBDL1	11

INPP5D	11
TRIM59	11
MMP1	11
CKAP2L	11
HIST1H4D	11
DLGAP5	11
ALAS2	11
MAD2L1BP	11
FBXO5	11
CCT2	11
E2F4	11
KLRG1	11
IGFBP3	11
CCNE2	11
FAM132B	11
SLC39A3	11
HIST1H2AG	11
NEUROD6	11
GYPA	11
RHOH	11
TRIM23	11
HBM	11
SMC4	11
GPRC5A	12
GZMA	12
GIMAP6	12
SOSTDC1	12
SFTA3	12
SELL	12
IGJ	12
CDC42EP5	12
SFTPD	12
IL7R	12
LRRN3	12
GIMAP2	12
SDR16C5	12
VGLL3	12
OSR1	12
TSPAN8	12
CEACAM6	12
MEST	12
TOX2	12
AQP4	12
PLAC8	12
HMCN1	12

CPA3	12
KLRK1	12
GZMB	12
CD97	12
MFAP4	12
LOC729680	12
FOXF2	12
COL6A3	12
LOC643733	12
FAM162B	12
C8orf4	12
OLFML1	12
RGS2	12
PLAT	12
SFTPA2	12
RGS18	12
SCGB1A1	12
TMEM100	12
RASSF2	12
CD69	12
P2RY14	12
SAMSN1	12
CYTIP	12
TRBC1	12
PTGS2	12
COLEC12	12
LAMP3	12
STAT4	12
ARHGAP15	12
IL2RB	12
KIAA1462	12
BTN3A3	12
ANKRD29	12
SFTPC	12
GPR171	12
CLDN18	12
SIDT1	12
CORO1A	12
SFTA2	12
NKG7	12
WFDC1	12
CD2	12
IFI27	12
HOXA5	12
MALL	12

AMICA1	12
C12orf75	12
MEF2C	12
IL32	12
SFTPB	12
NLRC5	12
CLIC3	12
CTHRC1	12
TRAT1	12
APOLD1	12
EGFL6	12
ITK	12
FAM65B	12
NPY	12
C16orf54	12
AOC3	12
GIMAP7	12
COL3A1	12
FOXF1	12
TGFBR2	12
LYVE1	12
HCLS1	12
SYNC	12
STK17B	12
C1QB	12
RAC2	12
TSPAN7	12
NRBF2	12
PAG1	12
TMEM71	12
COL6A6	12
CST7	12
SLC6A14	12
SLC22A4	13
LUM	13
ALDH1A1	13
SDPR	13
AKR1C3	13
NNMT	13
PLSCR4	13
GBP3	13
PDZK1IP1	13
FAM176A	13
HEMGN	13
HBB	13

CAT	13
SRGN	13
SERPINE2	13
CYBRD1	13
HBD	13
SLFN11	13
CD302	13
IFIT3	13
FAM210B	13
CNN3	13
PTGS2	13
AQP1	13
SLC40A1	13
ALDH8A1	13
C10orf116	13
TFPI	13
BICC1	13
FOSB	13
PKIG	13
SERPIND1	13
IFI27	13
C10orf10	13
LOC338758	13
SPTA1	13
GCLM	13
ZBTB16	13
LPIN2	13
RHAG	13
LINC00341	13
EPB42	13
TGFB1I1	13
SLC16A4	13
CTGF	13
SAMD9	13
ELTD1	13
PEAR1	13
MT2A	13
FBN1	13
BGN	13
COL8A1	13
ZNF23	13
HEBP1	13
COG1	13
SLC2A10	13
CTHRC1	13

KLF1	13
MAFF	13
COL3A1	13
SERPINA3	13
SFTPB	13
DNM3OS	13
GPR146	13
FOXF1	13
GRB10	13
C19orf77	13
UHRF2	13
ATP10D	13
TSPAN13	13
SELO	13
ENPEP	13
MMRN1	13
SLC14A1	13
CD59	13
MZB1	13
GADD45A	13
CEBPD	13
PTRF	13
SLIT2	13
FN1	13
AKR1C2	13
ACSM2A	13
ART4	13
FTH1	13
ST7	13
CYP8B1	13
GRAMD1C	13
NT5E	13
SAT1	13
AHSP	13
ZNF326	13
IRF1	13
NEK7	13
NRN1	13
YPEL3	13
ECE1	13
NEAT1	13
LIMS2	13
PRDX2	13
CSTA	14
S100A4	14

SLC7A5	14
FZD7	14
GREM1	14
CPVL	14
IMPA2	14
C5orf62	14
PYCARD	14
CRABP2	14
CYP1B1	14
ATIC	14
FAM129A	14
EMB	14
HAT1	14
CAPG	14
TGFBI	14
DEGS1	14
CTSK	14
MTHFD2	14
TMX1	14
GPNMB	14
HOXC6	14
SLC22A15	14
PHGDH	14
SFRP2	14
STAM	14
DAD1	14
LYPD3	14
TUBA1A	14
SHCBP1	14
IL18	14
S100A2	14
PLTP	14
RBM7	14
PRG2	14
CYBRD1	14
AHRR	14
RAB34	14
TAGLN	14
NAGK	14
KRT1	14
CEBPB	14
ETF1	14
PRPS1	14
PCNA	14
HS3ST3A1	14

STOML2	14
MRPL15	14
CFD	14
UQCRFS1	14
AZIN1	14
NMU	14
AAMP	14
PABPC3	14
RBPJ	14
APEX1	14
HLA.DMA	14
IARS	14
TMEM14C	14
CLIC1	14
GRN	14
TMEM45A	14
EIF3E	14
RPS4Y1	14
UBE2T	14
S100A11	14
ARHGEF6	14
CYB5R4	14
CKS2	14
PDIA6	14
CCNG1	14
KLHL21	14
CTDNEP1	14
C11orf75	14
LSM2	14
DEPDC1B	14
GPN2	14
FABP5	14
TSPO	14
EMP3	14
TIMP1	14
C1D	14
IPO4	14
VPS37A	14
NANP	14
EREG	14
KRT2	14
ALAS1	14
FST	14
OSTC	14
KLK5	14

RPL36	14
CTNNBIP1	14
VSIG10L	14
CDK4	14
PDCD10	14
COL6A3	14
VCAN	14
LOC152217	14
CD24	15
SCRN1	15
GREM1	15
C12orf75	15
CNTN3	15
SELM	15
SLC25A43	15
SPARC	15
CTHRC1	15
CAPG	15
PITX2	15
PPP3CA	15
MARCKS	15
F2RL1	15
RAB31	15
RNF128	15
TSPAN1	15
UGT8	15
PLS3	15
NQO1	15
PRSS8	15
HOXA5	15
MYH1	15
CTSK	15
SFRP2	15
CXCL14	15
HLA.DRA	15
ENPP2	15
MMP3	15
FOXF2	15
HOXC6	15
ATL1	15
TNNC2	15
MFAP2	15
FGF13	15
DDAH1	15
THY1	15

MEST	15
MGST1	15
WFDC1	15
LAMC1	15
STEAP1	15
HSD11B2	15
PFN2	15
FLRT3	15
LRIG3	15
ADAMDEC1	15
MYOZ1	15
ACTN3	15
ARMCX2	15
FZD2	15
MYLPF	15
GPR12	15
CALB1	15
SIM1	15
WBP5	15
CHRDL1	15
PLIN3	15
LPPR4	15
ABP1	15
DDIT4L	15
MIOX	15
RND3	15
COL15A1	15
SCN4A	15
IGJ	15
LOC645638	15
GPRC5A	15
SLIT2	15
LOX	15
ASB5	15
TCEAL7	15
LOC100506421	15
OLFML3	15
SKAP2	15
MYBPC1	15
CLRN3	15
RNF186	15
TMEM200A	15
C1orf106	15
TSPAN8	15
KCNS3	15

BICC1	15
SH3RF1	15
EPCAM	15
GPR160	15
AHCYL2	15
FRMD6	15
COL4A1	15
GDA	15
CXXC5	15
CYR61	15
KRTAP1.5	15
SERPINB7	15
MEF2C	15
LINC00520	15
PPAP2B	15
FSTL1	15
WASF1	15
PRSS23	15
CSTA	16
KRT77	16
SLC16A14	16
RAB38	16
S100A8	16
KRT14	16
KLF8	16
NAP1L3	16
WDR47	16
LOR	16
MMRN1	16
SPHKAP	16
KRT2	16
LCE2B	16
KRT15	16
ELOVL7	16
DSC3	16
FABP5	16
CTSL2	16
ELOVL4	16
SERPINA12	16
ZNF571	16
ZNF256	16
TCEAL4	16
TNFAIP8	16
C21orf91	16
CPVL	16

KRT5	16
LCE1B	16
MUCL1	16
KIF21B	16
C19orf59	16
FLG2	16
BEX5	16
KRT1	16
SLC22A15	16
GNA15	16
PRKAR2B	16
SBSN	16
DCD	16
SERPINB2	16
DSC1	16
FLG	16
PTPRZ1	16
HMGCR	16
ZNF682	16
ELMOD1	16
TAC1	16
FLJ45445	16
TUBB2B	16
EMB	16
FXYD6	16
NAP1L1	16
PEG3	16
SLC30A3	16
GJB6	16
TUBA1A	16
GPRASP1	16
LPL	16
PROK2	16
MCTP1	16
LY6D	16
NUDT11	16
NOP58	16
ZNF750	16
DLK1	16
SPATA22	16
CAPNS2	16
EGR2	16
PCP4	16
PCDH9	16
RTN1	16

SCEL	16
LPAR4	16
CCL27	16
CRISPLD1	16
RPS13	16
TUBB2A	16
LTA4H	16
ATRNL1	16
MNDA	16
LOC100507397	16
LANCL1	16
NOVA1	16
MDP1	16
C5orf47	16
RAN	16
WFDC5	16
ITM2A	16
DPY19L2P2	16
WIF1	16
BEX1	16
LOC100130476	16
GATA3	16
CTSG	16
SPATA6	16
EIF3H	16
EVI2A	16
DPYSL2	16
BNIP3	16
C16orf54	17
ELOVL7	17
CYTIP	17
RCSD1	17
SELL	17
LOC100506098	17
F13A1	17
PROM1	17
MGC24103	17
CD24	17
C1orf186	17
RNASE2	17
RNASE3	17
FAM150B	17
LOC285084	17
TRIM73	17
PIGR	17

PRAM1	17
HLA.DMB	17
MICAL1	17
LOC100132046	17
CECR1	17
GAPT	17
PARVG	17
LOC148145	17
LOC644656	17
CTF1	17
P2RY8	17
GJD3	17
CHKB	17
LOC202025	17
TMEM72	17
TRIM63	17
LOC100505501	17
SLC16A9	17
FAM135A	17
DPY19L2	17
TLR2	17
ARHGEF6	17
HLA.DMA	17
SYT6	17
TXNDC8	17
CD69	17
SYTL1	17
TMEM52B	17
NSUN7	17
ADCK1	17
RGL3	17
CSF2RB	17
SYK	17
LOC100505853	17
PLBD1	17
RRAGD	17
HMHA1	17
RNF150	17
DSERG1	17
KL	17
GUCY1A3	17
ERP27	17
USP44	17
SLC22A12	17
ENPP6	17

CLC	17
FAM155B	17
BEX2	17
IQCA1	17
PF4	17
ZNF335	17
LOC100130278	17
S100A12	17
BEX5	17
EPCAM	17
FBXW4P1	17
GPC4	17
GATA3	17
LCK	17
HLA.DPA1	17
SLC6A3	17
LOC100505878	17
THBS4	17
LHFPL3.AS2	17
ARRB1	17
C9orf66	17
CASQ2	17
CMTM2	17
ZNF721	17
LOC100130051	17
PRDM16	17
EVI2B	17
PRORSD1P	17
EFCAB12	17
TMEM216	17
KIAA1908	17
SNAI1	17
DNMT1	17
RASAL3	17
LRMP	17
LOC100506473	17
OR2B3	17
TCP10	17
CD247	18
GZMA	18
SCGB3A2	18
FREM2	18
NKG7	18
NELL2	18
ITK	18

GZMB	18
WIF1	18
CDH1	18
VAMP8	18
CCL4	18
CD2	18
CA2	18
NET1	18
MAL2	18
LAMP3	18
EPCAM	18
SLC25A39	18
IL32	18
PLS1	18
CFI	18
SCGB1A1	18
SLAIN1	18
AQP1	18
SFTPB	18
BCL2L1	18
SOCS2	18
TIAM1	18
DNAAF2	18
TCEAL2	18
TNF	18
CCT2	18
HERC5	18
ETS2	18
CKS2	18
FAM60A	18
CRISPLD1	18
FOS	18
KIF14	18
CD69	18
MFAP1	18
OXNAD1	18
UBE2S	18
EGFL6	18
IFNG	18
CYB5A	18
GLUL	18
SFTPD	18
LCK	18
TRAT1	18
GIMAP7	18

C4BPA	18
SLC38A5	18
USP44	18
TRBC1	18
SERINC5	18
GMNN	18
GPX3	18
CCNA2	18
TMSB15A	18
NAMPT	18
LRRN3	18
MAD2L2	18
CLIC2	18
SLC34A2	18
RHOU	18
ILF2	18
HAUS1	18
MSH2	18
PRKCQ	18
IDO1	18
MID1IP1	18
ICOS	18
CTNNAL1	18
RNF182	18
ARL6IP1	18
PNP	18
C1QBP	18
IL7R	18
MSL2	18
PGRMC1	18
FAM3B	18
CD3D	18
DUSP5	18
ZBED2	18
TAF9	18
ZBTB8OS	18
GPM6B	18
BEX4	18
ID1	18
GCH1	18
RASGRP1	18
C7	18
LAP3	18
RAB39B	18
NR4A2	18

ASNA1	18
CXCL10	18
ICAM2	18
BEX1	19
STXBP1	19
ENO2	19
TUBA1A	19
PRICKLE2	19
NAP1L3	19
MLLT11	19
SLC7A5	19
LARP6	19
THY1	19
EPDR1	19
TCEAL2	19
NGFRAP1	19
ATP6V0E2	19
STK39	19
SYNGR3	19
CNRIP1	19
ARNT2	19
IRS2	19
IRF2BPL	19
CLSTN1	19
WASF1	19
MTCH1	19
SPOCK1	19
PRRT3	19
MAP7D2	19
PGAM1	19
MYH10	19
ZNF580	19
TMEM178A	19
MRFAP1	19
HSPA1A	19
GPRASP2	19
PRR24	19
MKRN1	19
TIMP3	19
BEX2	19
HIF1A	19
EFEMP2	19
FAM171A1	19
NUAK1	19
GDI1	19

ZNF764	19
APBA2	19
KLC2	19
TOX2	19
TUBB2B	19
PFKFB3	19
PIP5K1C	19
HEG1	19
RGL1	19
FTH1	19
PTGDS	19
HBD	19
TUBB2A	19
MRAS	19
PCDH17	19
LOC100129550	19
MAL	19
UBE4A	19
PNMAL1	19
NARS	19
FAM127A	19
TUFT1	19
MNT	19
CUTA	19
THBS2	19
PAPD7	19
RPL41	19
ADM	19
SORCS2	19
SLC25A44	19
SYNDIG1	19
CDR2	19
YPEL5	19
LOC284889	19
TCTA	19
LDOC1	19
AMMECR1L	19
HOXC6	19
BANP	19
LBH	19
NEFM	19
INPP5J	19
IPCEF1	19
NACAD	19
NEFL	19

UCHL1	19
CACNA2D2	19
APLP1	19
ZCCHC24	19
C10orf35	19
KBTBD11	19
RTN1	19
C4orf48	19
PNMA1	19
KIF5C	19
FKBP1B	19
TP53INP2	19
EIF5A2	19
EMX2	20
PDZK1IP1	20
ATP6V0A4	20
RASGRP1	20
LOC100505761	20
ABP1	20
STAP1	20
SLC16A4	20
FMO1	20
CRYM	20
KL	20
PP7080	20
LRRC19	20
SLC5A12	20
TINAG	20
SLC13A1	20
ACE2	20
UGT2A3	20
FAM46C	20
ATP6V1G3	20
CTXN3	20
TMEM27	20
CLDN8	20
P2RY8	20
SERPINI1	20
LRP2	20
TWIST1	20
FCRL1	20
PCK1	20
MGAM	20
GPR18	20
PLD6	20

CDC42SE2	20
TCL1A	20
SLC22A6	20
HIST1H2AG	20
SLC6A13	20
OLFM4	20
CYS1	20
HEPACAM2	20
LRMP	20
SLC4A4	20
IGSF11	20
SLC3A1	20
EGF	20
SP140	20
UBE2QL1	20
FAM134B	20
EID3	20
CLRN3	20
MIOX	20
NEIL2	20
CALB1	20
CLEC18A	20
FXYD2	20
ITGA4	20
MS4A1	20
42979	20
SLC7A9	20
ENDOD1	20
ZNF490	20
BBOX1	20
ZYG11A	20
SOWAHA	20
NPY	20
DDC	20
FHOD3	20
CHPT1	20
P2RY10	20
GDA	20
PAQR7	20
SCN2A	20
GNPDA2	20
HPD	20
RASA4	20
COL15A1	20
DPY19L2P2	20

SSX2IP	20
TMEM52B	20
CYFIP2	20
CDH16	20
GBA3	20
SCN3A	20
LOC727820	20
FLJ22763	20
COX7B	20
ZNF439	20
IGJ	20
HOXD10	20
PARM1	20
CD19	20
LOC401522	20
LENG8	20
TNFRSF11B	20
ZNF367	20
ZNF675	20
MIRLET7DHG	20
HOXA10	20
PDZK1	20
LOC100288675	20
GPX3	21
ALDH2	21
CTSH	21
FTL	21
DSP	21
IFITM3	21
UBC	21
AOX1	21
NPC2	21
TIMP2	21
KMO	21
BSG	21
PEPD	21
COX1	21
RPL36A	21
HHEX	21
TMEM66	21
APLP2	21
LAMB2	21
DIO1	21
YIF1A	21
HEXB	21

TMEM176A	21
SLC7A7	21
FOS	21
BLVRB	21
PTTG1IP	21
RPS29	21
GCNT2	21
42796	21
PSAP	21
QPRT	21
EFHD1	21
ATP6V1A	21
COX2	21
CXXC5	21
TCF4	21
ASAH1	21
SLC25A1	21
ISCU	21
HLA.C	21
BLNK	21
TYK2	21
FTH1P5	21
RBPMS2	21
PKIG	21
RPL39	21
CD81	21
GCDH	21
RPS7	21
PTMA	21
NDUFA11	21
CPQ	21
APOM	21
YPEL5	21
SERF2	21
HLA.DMA	21
TAGLN	21
RAMP1	21
GPX1	21
RPL9	21
FCGRT	21
FXYD2	21
SUMF1	21
NPIP	21
TUBB2A	21
DFNA5	21

LAMTOR1	21
HLA.DMB	21
RPL41	21
MRFAP1	21
HLA.A	21
PYCARD	21
PDZK1	21
MPP1	21
MLF2	21
CCND1	21
ATP6V0C	21
ADM	21
HOGA1	21
PAPSS2	21
RPL3	21
JAZF1	21
ACAA1	21
HLA.DPA1	21
SIDT2	21
CAPG	21
RPL24	21
HMOX1	21
RPL11	21
UQCRH	21
RAB11FIP5	21
RAB13	21
ARRDC4	21
SH3BGRL	21
PDLIM1	21
FAM151A	21
RABAC1	21
VPS51	21
HSPB1	21
MIR155HG	22
CYTIP	22
EVI2A	22
RAB33A	22
PSTPIP2	22
SNX10	22
ABRACL	22
BCL2A1	22
LYSMD2	22
NEFH	22
ITGAL	22
HENMT1	22

SLC12A5	22
MLLT11	22
DOCK10	22
TMEM170B	22
CORO1A	22
TTC7B	22
FADS1	22
KCNAB2	22
COMMD9	22
SRGN	22
SYT11	22
IGSF6	22
TNFRSF1B	22
LGALS1	22
RCSD1	22
P2RY8	22
TCIRG1	22
ZNF804A	22
AMPD2	22
CCL4	22
CUX2	22
LY96	22
LPIN1	22
NPY	22
FAM220A	22
SACS	22
CD83	22
RFPL1.AS1	22
ACAT2	22
RASSF2	22
NABP1	22
GBP5	22
IL1B	22
WDR47	22
MYH1	22
GFI1	22
GCSAML	22
AKR1B1	22
GLIPR1	22
ZNF25	22
NETO2	22
ACYP2	22
CNDP1	22
C1orf216	22
PREX1	22

GTSF1	22
SAMSN1	22
SLC31A2	22
EVI2B	22
ALAS1	22
HAMP	22
COTL1	22
IL8	22
ACOT4	22
GMFG	22
SLAIN1	22
RASGRP1	22
SCN2A	22
C3orf80	22
CA10	22
RYR1	22
LOC285628	22
CCDC92	22
CRTAM	22
PSMB9	22
C20orf24	22
SUCNR1	22
C9orf91	22
SGTB	22
FBXO41	22
CTPS1	22
LDHA	22
SNAP91	22
PTGER2	22
MYO5A	22
JAKMIP2	22
LST1	22
CNP	22
GZMB	22
TEX30	22
SASH3	22
CD63	22
CNIH3	22
SV2A	22
VAMP7	22
TCEB1	22
PAG1	22
CREG2	22
HOXC6	23
TFPI2	23

GPX8	23
DKK1	23
MGC24103	23
PAQR7	23
MMP1	23
LOC375295	23
NANOG	23
PTX3	23
EMX2	23
ZNF273	23
LENG8	23
LOXL1.AS1	23
WNT5A	23
RAI14	23
WDR63	23
WRN	23
ZNF662	23
LOC100132046	23
DSEL	23
TUFT1	23
LOC100288152	23
ARSJ	23
SERPINB2	23
FAT1	23
MIRLET7DHG	23
OSR2	23
CCDC114	23
SPIN4	23
CELSR2	23
PTPN13	23
FANK1	23
HOXD8	23
CRIM1	23
CRISP3	23
LOC100506718	23
LOC400043	23
TPBG	23
TTC21B	23
CNTN3	23
SLC7A11	23
DCDC5	23
TTC18	23
DZIP1	23
AASS	23
DNAH7	23

DLG5	23
LIMS3	23
NSUN7	23
PRRT3	23
LOC728093	23
TMEM158	23
ZNF528	23
LOC100507312	23
SPAG6	23
SPATA1	23
CD109	23
ERRFI1	23
FGF2	23
ID4	23
STK36	23
ALCAM	23
LRRC37A4P	23
PDZK1	23
ZNF618	23
TMEM45A	23
COL5A1	23
SMARCD2	23
OXTR	23
BNC2	23
CCNA1	23
TWIST1	23
LOC100131541	23
RBBP5	23
LYPD1	23
ETAA1	23
MOB1B	23
PRKD1	23
GREM1	23
CCDC113	23
KIAA1598	23
KIF4A	23
THBS2	23
LOC100506114	23
PUS7	23
TMEM98	23
DNALI1	23
THBS1	23
GINS1	23
CABYR	23
PABPC4L	23

LOC100130429	23
STOML3	23
LOC284023	23
CDR1	23
ADM	23
EZH2	23
LRRC6	23
CADPS2	23
CAPN3	24
SCN3B	24
MYL1	24
ZBTB16	24
C8orf22	24
MYH2	24
OLIG1	24
ATP1A2	24
LOC285812	24
TMOD4	24
GPM6A	24
SYT17	24
MYRIP	24
SYT1	24
TSPAN7	24
CASQ1	24
WDR47	24
SH2B2	24
MYBPC1	24
IL17D	24
ATP6V0A1	24
OMG	24
BCL6	24
SYBU	24
PDE2A	24
TNNC2	24
PLEKHM2	24
ARRDC2	24
TMEM170B	24
LOC729680	24
TNNI2	24
AQP4	24
UBE2QL1	24
SNAI3	24
NEB	24
ATP2A1	24
ELMOD1	24

RYR1	24
MAP2	24
WBP2	24
VSNL1	24
PHYHIP	24
MAFB	24
LOC100190986	24
ERMN	24
DYSF	24
BCHE	24
TUBA4A	24
TM6SF1	24
MYBPC2	24
ABCB4	24
NEFL	24
KRT5	24
SLC6A1	24
PTPRZ1	24
FAM49A	24
NRN1	24
CAMK2G	24
NR1D2	24
CD40	24
TMCC3	24
TRDN	24
TNNT3	24
PDE4DIP	24
SCN1B	24
MYOZ1	24
TUBB2A	24
SLC38A11	24
RUNDC3A	24
ZNF438	24
CNDP1	24
PNPLA6	24
AMPH	24
ITIH1	24
TAZ	24
AQP9	24
SOX8	24
KMO	24
TMEM38A	24
KIAA1737	24
YPEL4	24
DUSP8	24

SNCA	24
SNAP25	24
BSN	24
RTN2	24
TSC1	24
RSPO3	24
ZEB2	24
STMN4	24
MYH1	24
UCP3	24
ZNF25	24
SCN4B	24
HMP19	24
MAN2A2	24
GABRA1	24
CYP4X1	24
IQSEC1	24
CARNS1	24
ESRP1	25
C15orf48	25
EPCAM	25
CA2	25
CASP6	25
MAL2	25
DSG2	25
ELOVL6	25
LIN28A	25
TOX3	25
APOA2	25
CEACAM7	25
HMGCS2	25
PANK1	25
RRAGD	25
ELOVL7	25
ADH1C	25
SFN	25
GUCY2C	25
HOOK1	25
CDH1	25
CLRN3	25
ADAMDEC1	25
LBR	25
LEFTY1	25
PRSS16	25
PGM2	25

FAM110C	25
SGPL1	25
SLC7A3	25
RHPN2	25
RAB25	25
CORO2A	25
AQP9	25
HPGD	25
SPINK5	25
SERINC5	25
PLA2G7	25
FBP1	25
GJB2	25
CYP2C18	25
C1orf131	25
DHCR24	25
DMKN	25
CYP3A5	25
ACOX2	25
APOC1	25
LINC00621	25
CLCA4	25
TNNT1	25
GCA	25
CPVL	25
NAT2	25
COBL	25
C10orf99	25
DSP	25
CSTA	25
C1orf106	25
LOC100505946	25
PKIB	25
SPINK1	25
PLG	25
LOC100996430	25
GCG	25
C10orf47	25
LRRN1	25
RARRES2	25
TRMT44	25
PIK3AP1	25
NANOG	25
SLC16A14	25
ORM1	25

ITLN1	25
ANXA10	25
PPP1R15B	25
CLDN1	25
PLXDC2	25
TNFRSF11A	25
IGF1	25
GPR19	25
EMB	25
ZIC2	25
MREG	25
PCK1	25
LRRC31	25
AFG3L2	25
LIN28B	25
EPB41L5	25
NOX1	25
CHMP4C	25
F9	25
RRP1B	25
PLEKHH1	25
FZD5	25
METTL7B	25
LINC00617	25
LINC00479	25
GOLT1A	25
CA14	25
SDCBP2	25
EPB41L3	26
ARRDC4	26
DMXL2	26
SLC38A6	26
SPP1	26
VCAN	26
PION	26
TFEC	26
ATP1B1	26
FIG4	26
LILRB1	26
DCSTAMP	26
RAB13	26
AIF1	26
TNFAIP6	26
LEFTY1	26
C1orf61	26

DAB2	26
OLR1	26
PLEK	26
KIAA1199	26
PLA2G7	26
GSN.AS1	26
HS3ST2	26
CD83	26
FCGR1B	26
GRPR	26
PTAFR	26
GAL	26
LAIR1	26
WDR11	26
CD300LF	26
FAM70A	26
PPAP2B	26
SLC7A7	26
ADAMDEC1	26
CXXC5	26
CXorf30	26
CYP26A1	26
LPL	26
OGFRL1	26
CCR5	26
FTH1	26
COX3	26
ESRRG	26
TNFSF13B	26
LYZ	26
RPS4Y1	26
STMN4	26
SLC22A15	26
SCML2	26
CTBP2	26
CEACAM4	26
FGR	26
RPS16	26
IGSF6	26
ARMCX1	26
M1	26
GPR183	26
HLA.DMA	26
ENPP2	26
SEMA3C	26

MYO5C	26
PBOV1	26
SH2B2	26
LRP12	26
SLC6A12	26
EVI2A	26
PHYHIPL	26
ANXA2P2	26
CD33	26
HIF1A	26
WBP5	26
PSMD6.AS2	26
PAPSS1	26
VIM	26
RAB42	26
BCL11A	26
MOB3B	26
DPYSL2	26
TTBK1	26
TUBB2B	26
ASPH	26
SLC22A4	26
WNT2	26
DNAH7	26
LOC100505478	26
CLEC7A	26
LOC285847	26
PSG6	26
OR10H3	26
TRHDE	26
ME1	26
CD68	26
HLA.DPB1	26
NPR3	26
PILRA	26
HLA.DPA1	26
SYT1	26
FPR3	26
SERPINB2	27
C15orf48	27
GPNMB	27
OSR2	27
S100A4	27
FAM129A	27
MFAP5	27

SQRDL	27
MAP2K3	27
HSPB6	27
CFD	27
TSPO	27
SBSN	27
PRRX1	27
PLEK2	27
GREM1	27
DSC1	27
KRT1	27
KRT2	27
COL15A1	27
COL6A3	27
FXYD3	27
SPINK4	27
DSG1	27
TPSAB1	27
CEBPB	27
SFN	27
CREB3L1	27
MB	27
FLG	27
GUCA2B	27
LGALS3	27
TUFT1	27
LIF	27
CD36	27
LOC150381	27
SYNPO2L	27
AHSP	27
SDR16C5	27
MMP3	27
FITM1	27
C2orf88	27
PLA2G2A	27
MXRA5	27
S100P	27
FAM132B	27
FAS	27
CEACAM7	27
CEACAM6	27
BDKRB2	27
TMEM173	27
PLP2	27

MYL2	27
CDX2	27
CLCA4	27
HIST1H1C	27
HBD	27
MICALCL	27
C10orf99	27
RETNLB	27
LRRN4CL	27
BEST2	27
PPDPF	27
MYL3	27
CLCA1	27
CDKN2C	27
IL20RB	27
GPRC5A	27
TFF1	27
FHL2	27
HEPACAM2	27
PRSS3	27
EN1	27
VDR	27
SLC26A3	27
GYPC	27
ITLN1	27
PPP1R14C	27
TNS1	27
PLEC	27
ASB2	27
MYH7	27
CD44	27
LOC100506621	27
CALML5	27
TPSB2	27
IGFBP3	27
PI3	27
MUC2	27
DEGS2	27
NFE2	27
MS4A12	27
MUCL1	27
MICAL2	27
GPA33	27
CD97	27
CLCA2	27

MIR22HG	27
PTPRH	27
TMEM54	27
C5AR1	28
AGR3	28
PLAC8	28
MARCKS	28
C15orf48	28
ACSL5	28
RSAD2	28
CLDN7	28
XK	28
FOXF1	28
CXCL3	28
CXCL16	28
SLC26A2	28
BCL2A1	28
CCL20	28
CCL18	28
AGR2	28
PRR15	28
TJP3	28
MLPH	28
LHFPL2	28
MBOAT1	28
EPCAM	28
SFTPA2	28
TFF3	28
GPC4	28
ADAMDEC1	28
SPRED1	28
TOR3A	28
CCDC114	28
IQGAP1	28
C7orf57	28
CLDN23	28
LYN	28
GALNT12	28
CEP55	28
SLC41A2	28
MEP1A	28
IGSF6	28
MS4A7	28
PIGR	28
KIF4A	28

STIP1	28
NCEH1	28
SCG5	28
SNTN	28
CCL8	28
KRT8	28
SLC7A11	28
OPN3	28
MYADM	28
CEACAM7	28
PTGS2	28
RAVER1	28
IL8	28
PLA2G10	28
DUSP5	28
NXPE1	28
HK2	28
SCGB2A1	28
IL7R	28
C19orf59	28
THEMIS2	28
FEM1C	28
PRCC	28
COPG1	28
CXCL1	28
TRANK1	28
GLIPR2	28
ATP13A3	28
CCDC78	28
GPRC5A	28
PLA2G7	28
SHROOM3	28
VPS41	28
LENG8	28
TMEM2	28
MMP12	28
UPP1	28
CCL2	28
LACC1	28
YIPF3	28
CDK7	28
TLR2	28
PRSS16	28
AP3B1	28
MS4A12	28

RHPN2	28
CTGF	28
LGALS4	28
MXD1	28
CORO2A	28
TREM2	28
ZSWIM6	28
BAX	28
C20orf85	28
CLCA1	28
SLC27A3	28
BIRC3	28
DSC2	28
BCL11A	29
RPL11	29
RPS29	29
RPL36A	29
RPS13	29
RPL32	29
RPL29	29
RPL41	29
HSP90AA1	29
TXNIP	29
LOC100507303	29
LOC100131564	29
RPS18	29
RPLP0	29
TPT1	29
OVGP1	29
SKP1	29
NR4A2	29
IRS2	29
YPEL5	29
PPP3CA	29
SNHG6	29
RPS25	29
MXI1	29
PFDN5	29
RPSA	29
RPL9	29
RPL39	29
UBC	29
RPL19	29
HMGB1	29
RPL21	29

RPS7	29
FAM13B	29
PRPS2	29
MLL5	29
RPS5	29
ANKRD36B	29
CRISP3	29
UBA52	29
RPL8	29
PNISR	29
R3HDM2	29
MYCBP2	29
SCAF8	29
LOC100507397	29
USP1	29
CXCR4	29
HIF1A	29
COX1	29
EEF2	29
PTMA	29
PLEKHG1	29
RPS3A	29
RPL24	29
RPL6	29
RPL30	29
EEF1B2	29
ND2	29
NARS	29
RPS3	29
DAPL1	29
MRFAP1	29
RAB11FIP2	29
TRAPPC8	29
TSPAN13	29
OPHN1	29
DNAH5	29
RPL26	29
MAP1LC3B	29
ARHGEF3	29
FOS	29
EIF3E	29
RPL34	29
RPS19	29
RPL4	29
SLC25A3	29

SCN1A	29
PTGES3	29
EEF1G	29
RPL36	29
RPL14	29
COX2	29
DACH2	29
RPS20	29
SMG1	29
PTPRT	29
FBXW7	29
RPL17	29
RGS2	29
EXTL2	29
LOC728093	29
FAM154B	29
RPL12	29
DPM1	29
RPS10	29
RPL27	29
ZNF273	29
ICAM2	29
FAU	29
LPL	30
MRC1	30
TUBB6	30
HLA.DRA	30
EMCN	30
TNNC1	30
GPX3	30
MNDA	30
NCF2	30
PCOLCE2	30
FCGR2B	30
ANXA3	30
AQP1	30
CCL2	30
RNASE6	30
LRRK2	30
SLC7A7	30
CDH5	30
CRNDE	30
C8orf4	30
CLIC4	30
FCER1G	30

TYRP1	30
FGR	30
HCK	30
GPR116	30
FABP4	30
EDNRB	30
RIT1	30
KDR	30
USP6NL	30
CAPG	30
LYN	30
CXCL16	30
PECAM1	30
OLR1	30
KMO	30
C5AR1	30
KL	30
CLIC2	30
CD36	30
MAMDC2	30
SOX7	30
FGD5	30
MS4A7	30
MPEG1	30
SFTA2	30
SLCO4C1	30
PLEKHG1	30
TFEC	30
ARHGAP24	30
MS4A4A	30
SESTD1	30
COLEC12	30
LAMP3	30
SWAP70	30
ESAM	30
TLR1	30
INMT	30
MGP	30
HMCN1	30
42796	30
SCGB1A1	30
NCEH1	30
CALCRL	30
PHF16	30
RBP7	30

PEBP4	30
GPR34	30
ECSCR	30
SLC26A9	30
PPP1R14A	30
CYYR1	30
THBD	30
GPR137B	30
F13A1	30
DARC	30
LAMB1	30
LAMC1	30
NEXN	30
CD93	30
FCGR1B	30
RAB31	30
GAPT	30
ALOX5	30
PROS1	30
NPC2	30
STEAP4	30
HLA.DQB1	30
C21orf7	30
CCDC50	30
ARRDC3	30
MME	30
ZNF624	30
UAP1L1	30
C7	30
ACP5	30
LYVE1	30
IL33	30
ABLIM3	30
